# EAT-Lancet diet and risk of metabolic dysfunction-associated steatotic liver disease and other liver chronic diseases: a large prospective cohort study in the UK Biobank

**DOI:** 10.3389/fnut.2025.1589424

**Published:** 2025-07-31

**Authors:** Shi-Yi Wu, Ya-Cong Bo, Ze-Yang Li, Xing-Yue Hu, Yang-Yang Ning, Jia Huang, Jun-Xi Zhang, Yong-Jian Zhu, Zeng-Li Yu, Hong-Yan Liu

**Affiliations:** ^1^Department of Medical Genetics, Henan Provincial People's Hospital, People's Hospital of Zhengzhou University, Zhengzhou, China; ^2^College of Public Health, Zhengzhou University, Zhengzhou, China; ^3^School of Public Health, Imperial College London, London, United Kingdom; ^4^NHC Key Laboratory of Birth Defects Prevention and Henan Key Laboratory of Population Defects Prevention, Zhengzhou, China; ^5^Department of Cardiology, The First Affiliated Hospital of Zhengzhou University, Zhengzhou, China

**Keywords:** EAT-Lancet diet, polygenic risk score, metabolic dysfunction-associated steatotic liver disease, chronic liver disease, gene-diet interaction

## Abstract

**Background and aim:**

As a newly recommended healthy dietary blueprint, the EAT-Lancet diet emphasizes both environmental sustainability and human health. However, its impact on chronic liver diseases remains unclear. This study examined the influence of the EAT-Lancet diet on the risk of metabolic dysfunction-associated steatotic liver disease (MASLD) and other chronic liver diseases.

**Methods:**

Our study included 160,394 UK Biobank participants who completed 24-h dietary assessments between April 2009 and June 2012, from which EAT-Lancet diet scores were calculated. The Cox proportional hazards models were used to estimate hazard ratios (HRs) with 95% confidence intervals (CIs) for the primary outcome (MASLD) and secondary endpoints, including cirrhosis, liver cancer, and other liver diseases.

**Results:**

A total of 1,727 cases of MASLD, 602 cases of liver cirrhosis, 103 cases of liver cancer, and 2,053 cases of other liver diseases were identified over a median follow-up period of 13.3 years. Using the lowest tertile as the reference, the highest EAT-Lancet diet index group demonstrated a 33% reduction in MASLD incidence (HR:0.67, multivariate 95%CI: 0.55, 0.80). In several secondary outcome measures, similar associations were also observed. Furthermore, the risk of MASLD was lowest among individuals with both higher EAT-Lancet dietary scores and lower genetic risk (HR = 0.52; 95%CI: 0.36–0.74), although no significant interaction was detected between the two groups.

**Conclusion:**

Adherence to the EAT-Lancet diet is associated with a reduced risk of chronic liver disease, independent of genetic factors.

## 1 Introduction

Suboptimal dietary factors increase the population disease burden and reduce the quality of life. Promoting healthy dietary patterns may serve as an effective strategy for preventing chronic diseases and supporting environmental sustainability (1, 2). In recent years, the EAT-Lancet diet, serving as a blueprint for healthy eating, has garnered significant attention (3). This dietary framework, designed by the EAT-Lancet Commission in 2019, is adaptable to different cultures, supports sustainability, and aims to bring about global changes in food production and waste while improving human health. It is mostly plant-based, advocating for increased consumption of fruits, vegetables, legumes, whole grains, and nuts, while limiting the intake of animal-derived foods, added sugars, and saturated fats. This framework strives to harmonize the relationship between healthy eating and the ecological environment, promoting sustainable development for both human health and the Earth’s ecology (2, 4). Evaluating whether adherence to this dietary framework can reduce the incidence of chronic diseases represents a critical research direction in public health. Current studies have demonstrated that this dietary pattern exhibits a lower association with the risk of developing conditions such as heart failure, lung cancer, and type 2 diabetes (5–7).

Globally, 4% of annual deaths are attributed to chronic liver diseases, representing a significant health burden (8). The most common of them is metabolic dysfunction-associated steatotic liver disease (MASLD), which impacts over 24% of the global population. In Europe, approximately one-quarter of individuals are affected by MASLD (9, 10). MASLD is not only a leading cause of hepatic disorders such as cirrhosis and hepatocellular carcinoma (11), but is also closely associated with T2DM, hyperlipidemia, obesity, and metabolic syndrome. It is a major driving factor for chronic liver disease and imposes substantial economic burdens while significantly reducing health-related quality of life (12, 13). Currently, there are no effective drug treatments for MASLD. Adopting a healthy lifestyle and adhering to dietary patterns in accordance with recommended guidelines serve as effective tools for preventing its development (14). Several studies have demonstrated the beneficial effects of healthy dietary patterns on metabolic dysfunction-associated steatotic liver disease (MASLD) (15–19). However, insufficient evidence currently exists regarding the impact of the EAT-Lancet diet on MASLD.

As another important factor affecting the progression of MASLD, genetic factors have attracted increasing attention in recent years. Genetic factors determine approximately half of liver steatosis and also determine the risk of metabolic diseases and hepatic fibrosis (20). Polygenic risk scoring (PRS) provides enhanced risk stratification by aggregating multiple susceptibility loci, thereby outperforming single-nucleotide polymorphism approaches in predicting incident MASLD (21). However, the impact of its interaction with diet on MASLD has not been thoroughly studied.

Therefore, we conducted this study to assess the relationship between adherence to the EAT-Lancet reference diet and the risks of MASLD, cirrhosis, liver cancer, and other chronic liver conditions and investigate whether these associations are modified by genetic risk.

## 2 Methods

### 2.1 Study population

The UK Biobank served as the data source for the present study. Specifically, during the baseline assessment period (2006–2010), the cohort enrolled 500,000 participants aged 39–70 years. All participants completed standardized physical examinations at one of the 22 dedicated assessment centers across England, Scotland, and Wales, supplemented by a touchscreen questionnaire and structured interviews conducted by trained nurses. Written informed consent was obtained from all participants prior to data collection.

**Figure 1:**
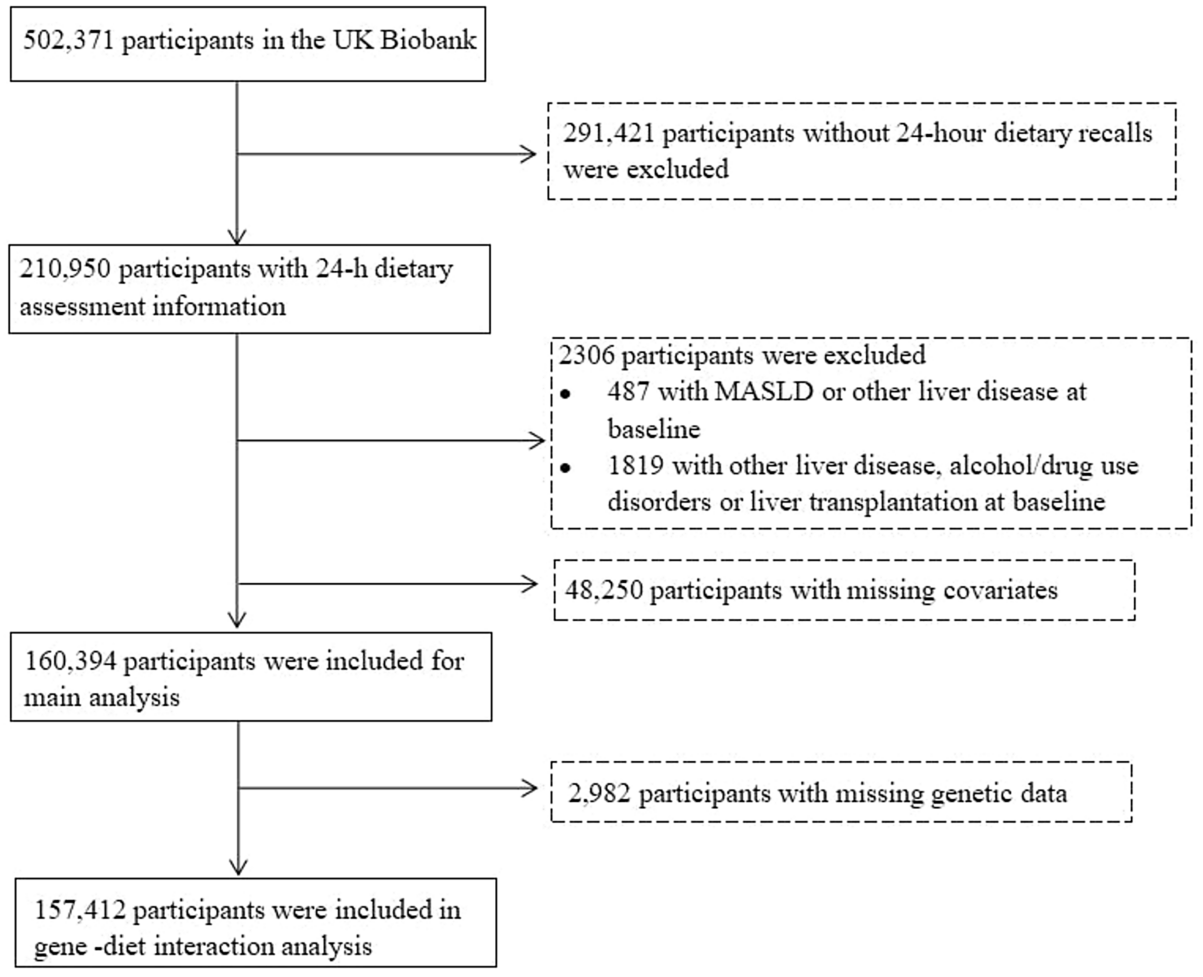
Flowchart of the study. MASLD, metabolic dysfunction-associated steatotic liver disease.

From an initial cohort of 502,371 individuals, the analytical cohort comprised 210,950 subjects with complete 24-h dietary recall data. The exclusion criteria included the following: (1) documented liver-related diseases or alcohol use (*n* = 509), (2) drug use disorders (*n* = 1,375), and (3) previous liver transplantation (*n* = 30) identified during baseline screening or historical records (Supplementary Table S3) (22, 23). The secondary exclusion criteria comprised the following: (1) MASLD (*n* = 195), (2) liver cirrhosis (*n* = 123), (3) liver cancer (*n* = 8, 4) other liver diseases (*n* = 463) detected at baseline assessment. Following rigorous adjustment for overlapping exclusions and missing covariate data, the final study population consisted of 160,394 eligible participants. Participants lacking genetic data (*n* = 2,982) were subsequently excluded from the PRS-diet interaction analysis, yielding a final analytical sample of 157,412 subjects for PRS–diet interaction modeling (Figure 1).

### 2.2 Dietary assessment

To quantify adherence to the EAT-Lancet diet, we developed a dietary score based on the methodology established by Knuppel et al., which represents one of the most widely applied healthy diet assessment tools. A binary scoring system was used to evaluate whether participants’ intake met the recommended upper and lower limits for each food category (24). The scoring system incorporated seven positively weighted components: whole grains, vegetables, fruits, legumes, nuts, fish, and unsaturated fats. In addition, seven inversely associated components were assessed: potatoes, dairy, eggs, pork, beef and lamb, poultry, and added sugars. Energy intake was standardized to 2,500 kcal/day for male participants and 2,000 kcal/day for female participants. The reference values for recommended intakes are assigned to each dietary component to determine the participants’ scores. A threshold value was set, and participants received 1 point if their score was below this value and 0 points otherwise. Higher cumulative scores reflected stronger alignment with healthier dietary patterns. The dietary index spanned a theoretical range of 0 (poorest adherence) to 14 (optimal adherence). The population distribution of dietary index scores is visualized in Supplementary Figure S1. For analytical purposes, scores were categorized into tertiles: lower adherence (0–10), moderate adherence (11), and higher adherence (12–14). More details on the calculations and food components are found in Supplementary Table S1.

### 2.3 PRS calculation

The PRS for MASLD is a weighted value obtained by weighting the number of risk alleles for each SNP. Our study combined 77 SNPs related to MASLD to determine the total PRS score. Specific details are provided in Supplementary Table S2. A higher score indicates a higher genetic susceptibility to MASLD. Participants who obtained scores were further stratified into three levels of genetic risk.

### 2.4 Outcome ascertainment

The primary outcome, MASLD, was defined according to the expert panel consensus statement using ICD-10 codes K76.0 (non-alcoholic fatty liver disease) and K75.8 (other specified inflammatory liver diseases) (22). It was identified based on hospital admission records and death registries. Secondary outcomes included liver cirrhosis, liver cancer, and other liver diseases (25). The ICD-10 codes used to ascertain these outcomes are detailed in Supplementary Table S4. The complete dates of inpatient data are as follows: October 2022 for England, August 2022 for Scotland, and May 2022 for Wales. Follow-up duration was censored at the earliest occurrence of (1) primary endpoint diagnosis, (2) loss-to-follow-up, (3) death, or (4) the study termination date (31 October 2022).

### 2.5 Assessment of covariates

Details regarding age and sex (male or female) were determined from self-reports. Covariates for sociodemographic factors and lifestyle were collected at baseline, including education (college or university degree), A levels/AS levels or equivalent, O levels/GCSEs or equivalent, CSEs or equivalent, NVQ or HND or HNC or equivalent, other professional qualifications (nursing, teaching, and none of the above), and income (<18,000, 18,000–30,999, 31,000–51,999, 52,000–100,000, and >£100,000 £/year). Using the Townsend score, the Townsend deprivation index is calculated from the residential postcode. Physical activity was grouped into low, moderate, or high. Smoking status and drinking status were categorized as current, former, or never. The body mass index (BMI) is calculated by dividing weight in kilograms by the square of height in meters (<25, ≥25 kg/m^2^). Information on whether participants had CVD, cancer, or diabetes was also collected.

### 2.6 Statistical analysis

Participant characteristics were summarized as mean ± standard deviation for continuous variables and frequency (percentage) for categorical variables. Multivariable-adjusted Cox regression models were implemented to estimate hazard ratios (HRs) with 95% confidence intervals (CIs) for associations between EAT-Lancet diet adherence and incident: (1) MASLD, (2) liver cirrhosis, (3) liver cancer, and (4) other liver diseases. The proportional hazards assumption was validated through Schoenfeld residuals (*α* = 0.05). Model 1 was adjusted to account for age and gender factors. On this basis, Model 2 further incorporated covariates including BMI, ethnicity, education qualifications, household income, smoking status, drinking status, physical activity, the Townsend deprivation index, and whether they suffer from CVD, cancer, and diabetes. Non-linear associations were characterized using restricted cubic splines with four knots placed at quintiles of the EAT-Lancet diet score distribution, implemented within the Cox proportional hazards framework. The joint effects of genetic predisposition (PRS tertiles) and dietary patterns (diet score tertiles) were assessed by creating a 3 × 3 matrix (9 combinations), using the highest-risk stratum (upper PRS tertile + lower diet score tertile) as the reference category. In a multivariable-adjusted model, hazard ratios (HRs) and 95% confidence intervals (CIs) were used to calculate the incidence of MASLD. Additive interaction was quantified using relative excess risk due to interaction (RERI) with delta method-derived confidence intervals, while multiplicative interaction was assessed through likelihood ratio tests comparing models with/without cross-product terms. Additionally, we performed subgroup analyses that were stratified by age (<65 and ≥65 years old), sex (female and male), smoking/drinking status (never and former/current), education (college or university and above, and other), and diabetes (no and yes).

We finally conducted two sensitivity analyses. First, we excluded participants who had been diagnosed with liver cirrhosis, liver cancer, MASLD, or other liver diseases within the first 2 or 4 years of the trial to evaluate the strength of the connection. Second, we attempted to account for missing covariates by performing multiple imputations.

A two-tailed test and a *p*-value of < 0.05 were considered significant. All analyses were performed using R software version 4.3.3.

## 3 Results

### 3.1 Baseline characteristics

Baseline characteristics are shown in Table 1. Among the 160,394 participants at baseline, the mean (SD) age was 56.2 years (7.97), and 83,870 (52.3%) were female. Overall, higher scores were associated with participants who are female, non-smokers or non-drinkers, have more free time for physical exercise, have higher education levels, and have a lower BMI.

**Table 1:** Baseline characteristics of 160,394 participants by the EAT-Lancet diet index.

Variables	EAT-Lancet diet index			
	Low (*N* = 88,687)	Moderate (*N* = 52,422)	High (*N* = 19,285)	Total (*N* = 160,394)
Age				
Baseline age, years, mean (SD)	55.8 (8.05)	56.7 (7.88)	56.5 (7.79)	56.2 (7.97)
Sex				
Female	41,732 (47.1%)	29,836 (56.9%)	12,302 (63.8%)	83,870 (52.3%)
Male	46,955 (52.9%)	22,586 (43.1%)	6,983 (36.2%)	76,524 (47.7%)
Ethnicity				
White	85,011 (95.9%)	50,378 (96.1%)	18,326 (95.0%)	153,715 (95.8%)
Non-white	3,676 (4.1%)	2,044 (3.9%)	959 (5.0%)	6,679 (4.2%)
Smoking status				
Never smoker	48,445 (54.6%)	30,734 (58.6%)	11,369 (59.0%)	90,548 (56.5%)
Previous smoker	32,063 (36.2%)	18,434 (35.2%)	6,876 (35.7%)	57,373 (35.8%)
Current smoker	8,179 (9.2%)	3,254 (6.2%)	1,040 (5.4%)	12,473 (7.8%)
Drinking status				
Never drinker	2,465 (2.8%)	1,512 (2.9%)	662 (3.4%)	4,639 (2.9%)
Previous drinker	2,450 (2.8%)	1,425 (2.7%)	635 (3.3%)	4,510 (2.8%)
Current drinker	83,772 (94.5%)	49,485 (94.4%)	17,988 (93.3%)	151,245 (94.3%)
Physical activity				
Low	17,930 (20.2%)	8,806 (16.8%)	2,725 (14.1%)	29,461 (18.4%)
Moderate	37,388 (42.2%)	22,671 (43.2%)	8,207 (42.6%)	68,266 (42.6%)
High	33,369 (37.6%)	20,945 (40.0%)	8,353 (43.3%)	62,667 (39.1%)
Household income				
Less than 18,000	13,271 (15.0%)	7,357 (14.0%)	2,714 (14.1%)	23,342 (14.6%)
18,000–30,999	20,911 (23.6%)	12,622 (24.1%)	4,497 (23.3%)	38,030 (23.7%)
31,000–51,999	25,417 (28.7%)	15,018 (28.6%)	5,485 (28.4%)	45,920 (28.6%)
52,000–100,000	22,325 (25.2%)	13,377 (25.5%)	4,990 (25.9%)	40,692 (25.4%)
Greater than 100,000	6,763 (7.6%)	4,048 (7.7%)	1,599 (8.3%)	12,410 (7.7%)
Education level				
College or university degree	37,908 (42.7%)	25,373 (48.4%)	10,656 (55.3%)	73,937 (46.1%)
A levels/AS levels or equivalent	12,035 (13.6%)	6,927 (13.2%)	2,502 (13.0%)	21,464 (13.4%)
O levels/GCSEs or equivalent	18,630 (21.0%)	9,935 (19.0%)	3,134 (16.3%)	31,699 (19.8%)
CSEs or equivalent	4,029 (4.5%)	1,761 (3.4%)	444 (2.3%)	6,234 (3.9%)
NVQ or HND or HNC or equivalent	5,282 (6.0%)	2,517 (4.8%)	767 (4.0%)	8,566 (5.3%)
Other professional qualifications e.g., nursing, teaching	4,082 (4.6%)	2,604 (5.0%)	858 (4.4%)	7,544 (4.7%)
None of the above	6,721 (7.6%)	3,305 (6.3%)	924 (4.8%)	10,950 (6.8%)
BMI (kg/m^2^)				
<25	29,469 (33.2%)	21,417 (40.9%)	9,688 (50.2%)	60,574 (37.8%)
≥25	59,218 (66.8%)	31,005 (59.1%)	9,597 (49.8%)	99,820 (62.2%)
Townsend deprivation index, mean (SD)	−1.56 (2.89)	−1.70 (2.80)	−1.49 (2.87)	−1.60 (2.86)
CVD				
No	64,867 (73.1%)	39,386 (75.1%)	15,117 (78.4%)	119,370 (74.4%)
Yes	23,820 (26.9%)	13,036 (24.9%)	4,168 (21.6%)	41,024 (25.6%)
Cancer				
No	82,305 (92.8%)	48,345 (92.2%)	17,703 (91.8%)	148,353 (92.5%)
Yes	6,382 (7.2%)	4,077 (7.8%)	1,582 (8.2%)	12,041 (7.5%)
Diabetes				
No	84,718 (95.5%)	50,457 (96.3%)	18,721 (97.1%)	153,896 (95.9%)
Yes	3,969 (4.5%)	1,965 (3.7%)	564 (2.9%)	6,498 (4.1%)

SD, standard deviation; BMI, body mass index; CVD, cardiovascular disease; NVQ, National Vocational Qualification; HND, Higher National Diploma; HNC, Higher National Certificate; GCSEs, General Certificate of Secondary Education; CSEs, Certificate of Secondary Education.

### 3.2 EAT-Lancet diet and incidence of chronic liver diseases

During a median follow-up period of 13.3 years, a total of 1,727 MASLD cases were reported. In age-sex adjusted models, participants in the moderate (HR = 0.68, 95%CI 0.62–0.75) and high (HR = 0.53, 95%CI 0.45–0.62) EAT-Lancet diet adherence tertiles demonstrated significantly lower MASLD risk compared to the lowest tertile (*p* < 0.001 for both). In the multivariate-adjusted Cox proportional hazards model, the hazard ratio (HR) was 0.67 (95%CI: 0.55–0.80; *p* < 0.001) for participants in the highest EAT-Lancet diet index group compared to the lowest group. Furthermore, each 3-point increase in the EAT-Lancet diet score was associated with a 29% reduction in the risk of MASLD (with HRs 0.71 [95%CI, 0.58–0.89]) (Table 2).

**Table 2:** Hazard ratios (95% confidence intervals) of MASLD, cirrhosis, liver cancer, and other liver diseases according to the tertiles of the EAT-Lancet diet index.

	Cases/person-years	Model 1		Model 2	
		HR (95% CI)	*p*-value	HR (95% CI)	*p*-value
MASLD					
tertile1	1,570/2,745,613	1.00 (Ref.)		1.00 (Ref.)	
tertile2	639/2,745,516	0.68 (0.62, 0.75)	**<0.001**	0.81 (0.73, 0.91)	**<0.001**
tertile3	178/2,745,353	0.53 (0.45, 0.62)	**<0.001**	0.67 (0.55, 0.80)	**<0.001**
Continuous (per 3 unit)		0.54 (0.49, 0.60)	**<0.001**	0.71 (0.58, 0.89)	**0.002**
Liver cirrhosis					
tertile1	546/2,752,353	1.00 (Ref.)		1.00 (Ref.)	
tertile2	232/2,752,256	0.74 (0.63, 0.86)	**<0.001**	0.87 (0.72, 1.04)	0.118
tertile3	55/2,752,093	0.51 (0.39, 0.68)	**<0.001**	0.55 (0.39, 0.78)	**<0.001**
Continuous (per 3 unit)		0.57 (0.48, 0.68)	**<0.001**	0.71 (0.57, 0.88)	**0.002**
Liver cancer					
tertile1	92/2,754,781	1.00 (Ref.)		1.00 (Ref.)	
tertile2	37/2,754,685	0.69 (0.47, 1.01)	0.057	0.74 (0.47, 1.14)	0.173
tertile3	3/2,754,521	0.17 (0.05, 0.53)	**0.002**	0.17 (0.04, 0.70)	**0.014**
Continuous (per 3 unit)		0.41 (0.26, 0.64)	**<0.001**	0.48 (0.29, 0.80)	**0.005**
Other liver diseases					
tertile1	1,644/2,745,329	1.00 (Ref.)		1.00 (Ref.)	
tertile2	858/2,745,232	0.85 (0.79, 0.93)	**<0.001**	0.94 (0.85, 1.04)	0.218
tertile3	285/2,745,069	0.80 (0.71, 0.91)	**<0.001**	0.85 (0.73, 0.99)	**0.038**
Continuous (per 3 unit)		0.76 (0.68, 0.84)	**<0.001**	0.85 (0.76, 0.96)	**0.009**

Model 1 was adjusted for age and sex.

Model 2 was adjusted for age, sex, ethnicity, Townsend deprivation index, educational level, BMI, physical activity, drinking status, smoking status, household income, CVD, cancer, and diabetes.

Bold value indicates Levels of significance: *p* < 0.05 (Cox regression model).

MASLD, metabolic dysfunction-associated steatotic liver; HR, hazard ratio; CI, confidence interval; CVD, cardiovascular disease.

For other chronic liver diseases, in the fully adjusted model, the highest EAT-Lancet diet adherence group exhibited a 45% reduction in cirrhosis risk (HR = 0.55, 95% CI 0.39–0.78), an 83% reduction in liver cancer risk (HR = 0.17, 95% CI 0.04–0.70), and a 15% reduction in other liver disease risks (HR = 0.85, 95% CI 0.73–0.99). Additionally, the hazard ratios (HRs) for cirrhosis, liver cancer, and other liver diseases were 0.71 (0.57–0.88), 0.48 (0.29–0.80), and 0.85 (0.76–0.96) per 3-point increase.

**Figure 2:**
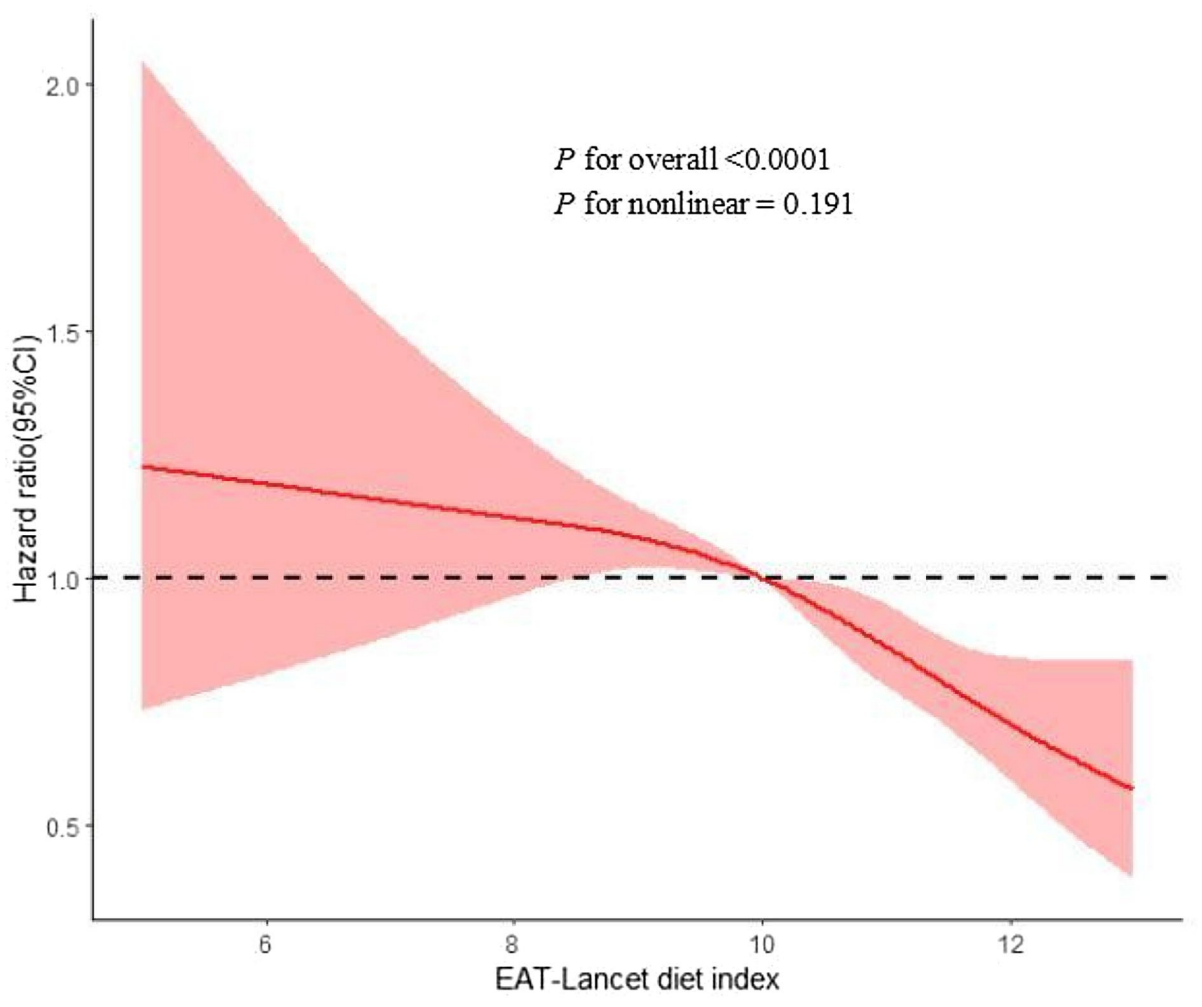
Restrict cubic spline for associations of the EAT-Lancet diet index with MASLD risk. Adjusted for age, sex, ethnicity, Townsend deprivation index, educational level, BMI, physical activity, drinking status, smoking status, household income, CVD, cancer, and diabetes. BMI, body mass index; CI, confidence interval; HR, hazard ratio; MASLD, metabolic dysfunction-associated steatotic liver; CVD, cardiovascular disease.

When examining the dose–response relationship, we observed a linear deviation in the association between the risk of MASLD and the EAT-Lancet diet (Figure 2, *p*-non-linearity > 0.05). Similar findings were also observed in liver cancer, cirrhosis, and other liver diseases (*p* for overall < 0.05, *p*-non-linearity > 0.05) (Supplementary Figures S2–S4).

### 3.3 The interaction of the EAT-Lancet diet and PRS on MASLD

**Figure 3:**
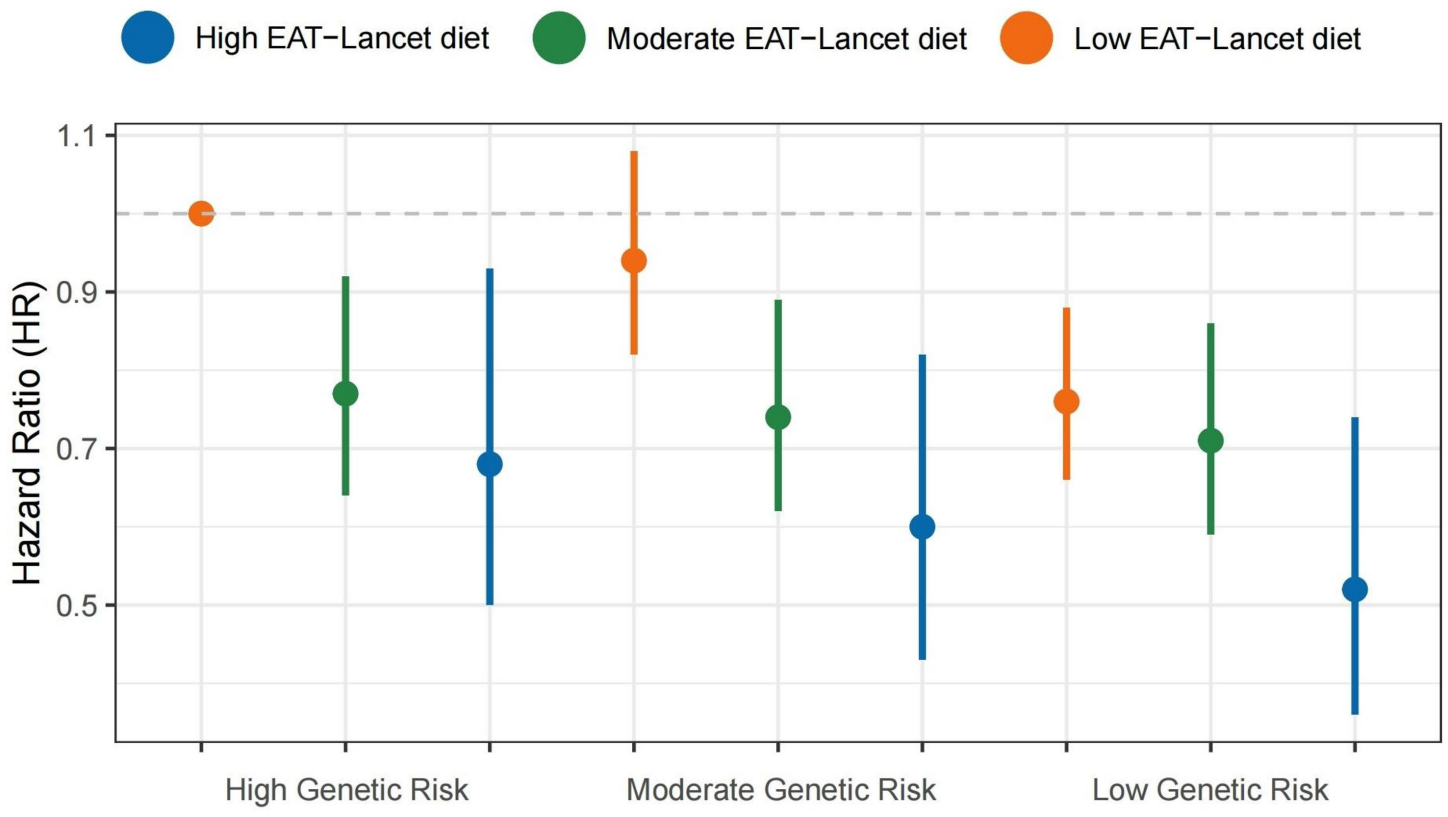
Combined effects of the EAT-Lancet diet index, genetic risk, and the risk of MASLD. MASLD, metabolic dysfunction-associated steatotic liver disease.

As detailed in Supplementary Table S5, participants in the highest PRS tertile exhibited 24% elevated risk of MASLD compared to the lowest tertile (HR = 1.24, 95%CI 1.10–1.40). Figure 3 illustrates the interactions between the genetic component and adherence to the EAT-Lancet diet. As genetic risk increases, the risk of MASLD onset decreases with higher EAT-Lancet diet scores. The protective effect was most pronounced in the low PRS/high diet adherence group (HR = 0.52, 95% CI 0.36–0.74, compared to the high PRS/low adherence group). However, neither additive nor multiplicative effects were observed (Table 3).

**Table 3:** Combined effects of the EAT-Lancet diet, PRS, and the risk of MASLD.

EAT-Lancet diet index	PRS*				*p* for interaction^++^
	Moderate		Low		
	RERI^+^	AP^+^	RERI	AP	
Moderate	−0.03 (−0.19, 0.24)	0.04 (−0.25, 0.32)	0.18 (−0.02, 0.38)	0.26 (−0.02, 0.53)	0.625
High	−0.03 (−0.33, 0.27)	−0.05 (−0.56, 0.47)	0.07 (−0.22, 0.37)	0.14 (−0.40, 0.69)	

AP, attributable proportion due to the interaction; CI, confidence interval; PRS, polygenic risk score; RERI, relative excess risk due to the interaction; CVD, cardiovascular disease.

Adjusted for age, sex, ethnicity, Townsend deprivation index, educational level, BMI, physical activity, drinking status, smoking status, household income, CVD, cancer, and diabetes.

*Defined by PRS: low (lowest tertiles), intermediate (second tertiles), and high (highest tertiles).

^+^To estimate the RERI and AP, the low EAT-Lancet diet index and the highest genetic risk (high PRS) groups were the reference categories.

^++^Likelihood tests were applied to test the significance of the interaction term by comparing the model with and without the interaction term.

### 3.4 Additional analyses

**Figure 4:**
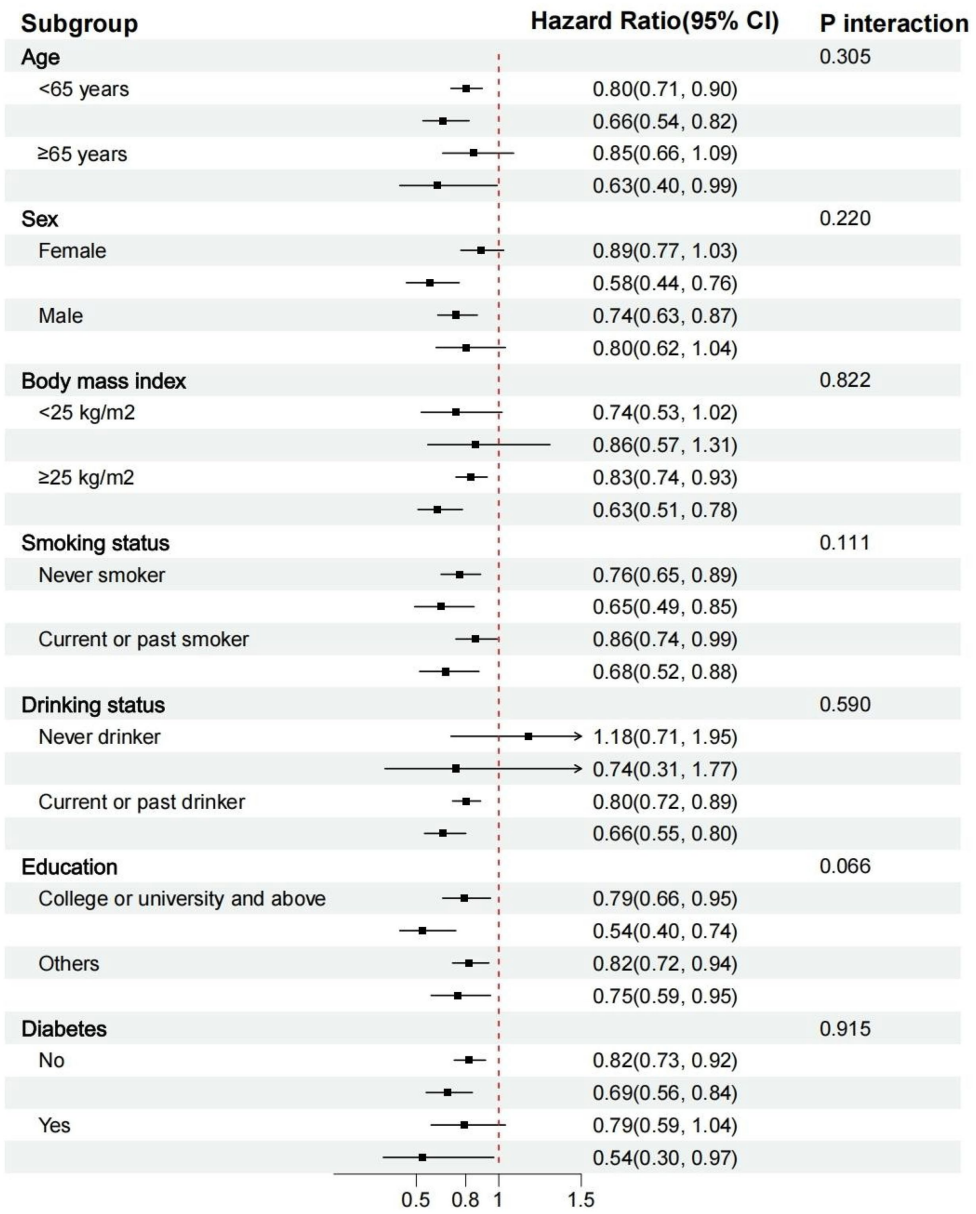
Stratified analyses of the association between the EAT-Lancet diet index and the risk of MASLD. MASLD, metabolic dysfunction-associated steatotic liver diseases.

Figure 4 displays the stratified analysis results. The risk of MASLD was not substantially impacted by variables, including age, gender, BMI, smoking or drinking, household income, education level, or diabetes (*p* for all interactions > 0.05). Supplementary Figures S5–S7 display a stratified analysis involving liver cirrhosis, liver cancer, and other liver diseases. We observed a significant interaction between the EAT-Lancet diet and liver cancer, as well as other liver diseases, among individuals of different genders (*p* for interaction < 0.05).

In sensitivity analysis, we excluded cases that occurred within the first 2 or 4 years, as shown in Supplementary Table S6. The results of multiple interpolations of covariates are shown in Supplementary Table S7.

## 4 Discussion

In this prospective study based on the UK Biobank, we observed that the EAT-Lancet diet index demonstrated significant protective effects against chronic liver disease. Compared to the lowest adherence group, individuals in the highest adherence group exhibited a 33% reduced risk of developing MASLD. Consistent results were obtained for secondary outcomes. Furthermore, the protective association persisted across genetic risk strata, supporting the EAT-Lancet diet as an evidence-based dietary recommendation for hepatic health promotion.

Although diet is recognized as a critical pillar in mitigating the development of chronic liver diseases, longitudinal studies investigating the association between the EAT-Lancet diet and the risk of MASLD remain limited. One study investigating this association yielded results consistent with our findings: Adherence in the highest quartile was associated with a 27% reduced risk of MASLD (26). However, no studies have yet explored its potential impact on other chronic liver diseases. A number of previous studies have pointed out that plant foods such as vegetables and beans are protective factors for MASLD (27). A cohort study from Tianjin found that compliance with animal-based or sugar-rich dietary patterns was positively associated with MASLD, whereas vegetable-rich dietary patterns showed no significant association with MASLD risk (28). Similarly, a UK Biobank study suggested that plant-based diets were associated with a 22 and 26% reduction in MASLD risk, respectively (16, 29). A randomized controlled trial has shown that adhering to a green-MED diet primarily composed of plant-based foods can halve the prevalence of MASLD (30). Our study corroborates this finding, indicating a positive relationship between the MASLD and the EAT-Lancet diet.

Genetic factors serve as another identified pathogenic risk, yet the interaction between these factors and diet has not yielded meaningful results in existing studies. The reason for this lack of interaction may be that the PRS constructed using a limited number of SNPs cannot accurately represent genetic risk. Constructing a more comprehensive PRS that covers a wider range of pathways and functions may better explain disease risk (31, 32). Although we used a larger number of SNPs (77) to construct the PRS compared to previous studies that included only five SNPs, no significant interaction was observed (33). Therefore, it is necessary to integrate more loci in the future to improve the prediction of individual genetic risk for MASLD. With the ongoing expansion of genome-wide association study (GWAS) sample sizes and advances in polygenic risk score (PRS) construction methods, future PRS models are expected to exhibit greater predictive accuracy and resolution. Reassessing the interactions between the EAT-Lancet dietary pattern and genetic susceptibility using these refined tools will be an important area for future investigation (34, 35). In our study, we found that both PRS and the EAT-Lancet diet can independently predict MASLD. This finding implies that individuals with different genetic risks should all pay attention to the quality of their diet. From the standpoint of public health, this dietary pattern is beneficial for patients with MASLD, regardless of genetic risk.

We first explored the connection between the EAT-Lancet diet and other chronic liver diseases (except for MASLD). Previous studies concentrated on the relationship between diet and liver-related diseases. For instance, Brazilian patients with liver cirrhosis consume more grains, rice, beans, and yogurt in their diet compared to American patients. This dietary pattern is linked to greater gut microbiota diversity and demonstrates a decreased hospitalization rate (36). A prospective study conducted as part of another Women’s Health Initiative found a negative correlation between adherence to a dietary pattern for reducing diabetes risk and the risk of liver cancer, as well as mortality from chronic liver disease. This dietary pattern aimed at reducing diabetes risk involves decreasing the total intake of red and processed meat, foods with a high glycemic index, sugar-sweetened beverages (SSBs), and dietary trans fats, while increasing the intake of cereal fiber, coffee, nuts, and polyunsaturated fatty acids (37). Furthermore, some studies have also found that higher baseline intakes of breakfast cereals, tea, fruits, and dietary fiber, coupled with lower intakes of red meat and processed meat, can reduce the risk of liver cirrhosis, liver cancer, and other related conditions (38).

The development of MASLD involves a wide range of pathophysiological mechanisms, starting with hepatocellular death, followed by inflammation and compensatory proliferation, and ultimately developing into different stages of liver fibrosis, cirrhosis, and hepatocellular carcinoma (39, 40). Currently, the biological mechanisms linking the EAT-Lancet diet and chronic liver diseases remain unclear, but diet also serves as a risk factor contributing to the burden of chronic liver diseases (41). High-fat and high-fructose diets can disturb the gut microbiota, inducing hepatic steatosis and inflammation and promoting tumorigenesis (41, 42). Insulin resistance is also a key triggering factor (43). Studies have shown that macronutrients such as saturated fatty acids (SFAs), trans fats, monosaccharides (sucrose and fructose), and animal proteins can regulate the accumulation of triglycerides and antioxidant activity in the liver, thereby affecting its sensitivity (44, 45). Additionally, necroptosis or pyroptosis of hepatocytes also drives the progression of MASLD (46). Similarly, certain biomarkers also play a profound mediating role in the relationship between healthy dietary patterns and disease risk (47, 48). Diet-associated proteins (e.g., FSTL3, STC1, and CD302 antigen) significantly mediate risk associations for various chronic disorders, including cardiovascular diseases and chronic respiratory diseases (49). Meanwhile, metabolic biomarkers related to the EAT-Lancet diet (specifically, triglycerides in HDL) exhibit a significant positive correlation with MASLD incidence, suggesting their potential involvement in mediating dietary influences on MASLD risk (33). After validation, these biomarkers may serve as comprehensive health indicators to compensate for biases inherent in conventional dietary assessments (47).

Our study’s strengths include its prospective design and large sample size. Our study has certain limitations as well. First, in large-scale population studies, the precise calculation of individual dietary intake is challenging. Dietary assessment relies on 24-h recall methods, which may be subject to recall bias and fail to adequately reflect long-term dietary habits. However, the dietary assessment metrics used in our primary analysis have been demonstrated to correlate with repeatedly measured dietary indices (50). Second, over half of the participants were eliminated due to incomplete 24-h dietary recall questionnaires, but this discrepancy can be clinically negligible (51). Third, currently, there is no unified standard for quantifying adherence to the EAT-Lancet diet. In addition to the methodology used in our study, the Stubbendorff scoring system is also commonly utilized. However, the Stubbendorff scoring method may not comprehensively account for the balance between health and environmental impacts (52). Nevertheless, both scoring systems demonstrated good adherence within the cohort. Some researchers have raised concerns that the Knuppel method may not serve as an optimal indicator for assessing adherence. However, Knuppel et al. clarified that their analysis was adjusted for energy intake, thereby enabling valid comparisons between participants with different adherence scores at equivalent energy intake levels (53). Fourth, although most of the confounding factors have been controlled, there are still some potential confounding factors. Finally, the majority of participants were white, which limited our study’s ability to accurately reflect the entire population.

## 5 Conclusion

Our study found that adherence to the EAT-Lancet diet is associated with a reduced risk of chronic liver diseases. Following this sustainable diet may attenuate the development of multiple chronic liver conditions, thereby substantiating the EAT-Lancet Commission’s global health recommendations.

## Data Availability

The raw data supporting the conclusions of this article will be made available by the authors without undue reservation.
