# Supplementary material for: EAT-Lancet diet and risk of metabolic dysfunction-associated steatotic liver disease and other liver chronic diseases: a large prospective cohort study in the UK Biobank
Source: Front Nutr. 2025 Jul 31;12:1589424. doi: 10.3389/fnut.2025.1589424 (PMC12350129; doi:10.3389/fnut.2025.1589424)
Supplement: Supplementary file 1 [file Table_1.docx]

EAT-Lancet diet score

**Figure S1.** Distribution of the EAT-Lancet diet index (range from 0 to 14 points) among this study sample in the UK Biobank.


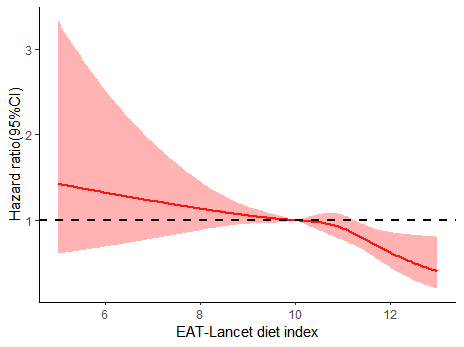


*P* for overall <0.0001

*P* for nonlinear =0.215

**Figure S2.** Restrict cubic spline for associations of EAT-Lancet diet index with cirrhosis risk. Adjusted for age, sex, ethnicity, Townsend deprivation index, educational level, BMI, physical activity, drinking status, smoking status, household income, CVD, cancer and diabetes.

Abbreviations: BMI, body mass index; CI, confidence interval; HR, hazards ratio; CVD, Cardiovascular Disease.


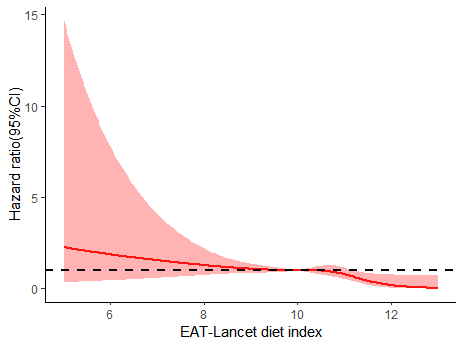


*P* for overall <0.0001

*P* for nonlinear =0.193

**Figure S3.** Restrict cubic spline for associations of EAT-Lancet diet index with liver cancer risk. Adjusted for age, sex, ethnicity, Townsend deprivation index, educational level, BMI, physical activity, drinking status, smoking status, household income, CVD, cancer and diabetes.

Abbreviations: BMI, body mass index; CI, confidence interval; HR, hazards ratio; CVD, Cardiovascular Disease.


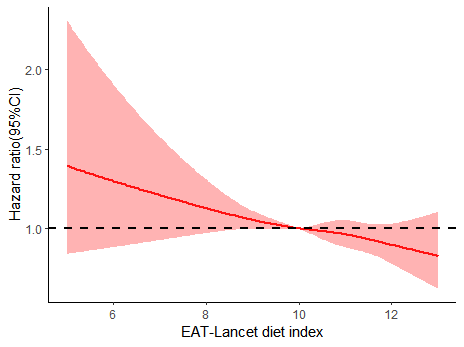


*P* for overall <0.0001

*P* for nonlinear =0.927

**Figure S4.** Restrict cubic spline for associations of EAT-Lancet diet index with other liver diseases risk. Adjusted for age, sex, ethnicity, Townsend deprivation index, educational level, BMI, physical activity, drinking status, smoking status, household income, CVD, cancer and diabetes.

Abbreviations: BMI, body mass index; CI, confidence interval; HR, hazards ratio; CVD, Cardiovascular Disease.


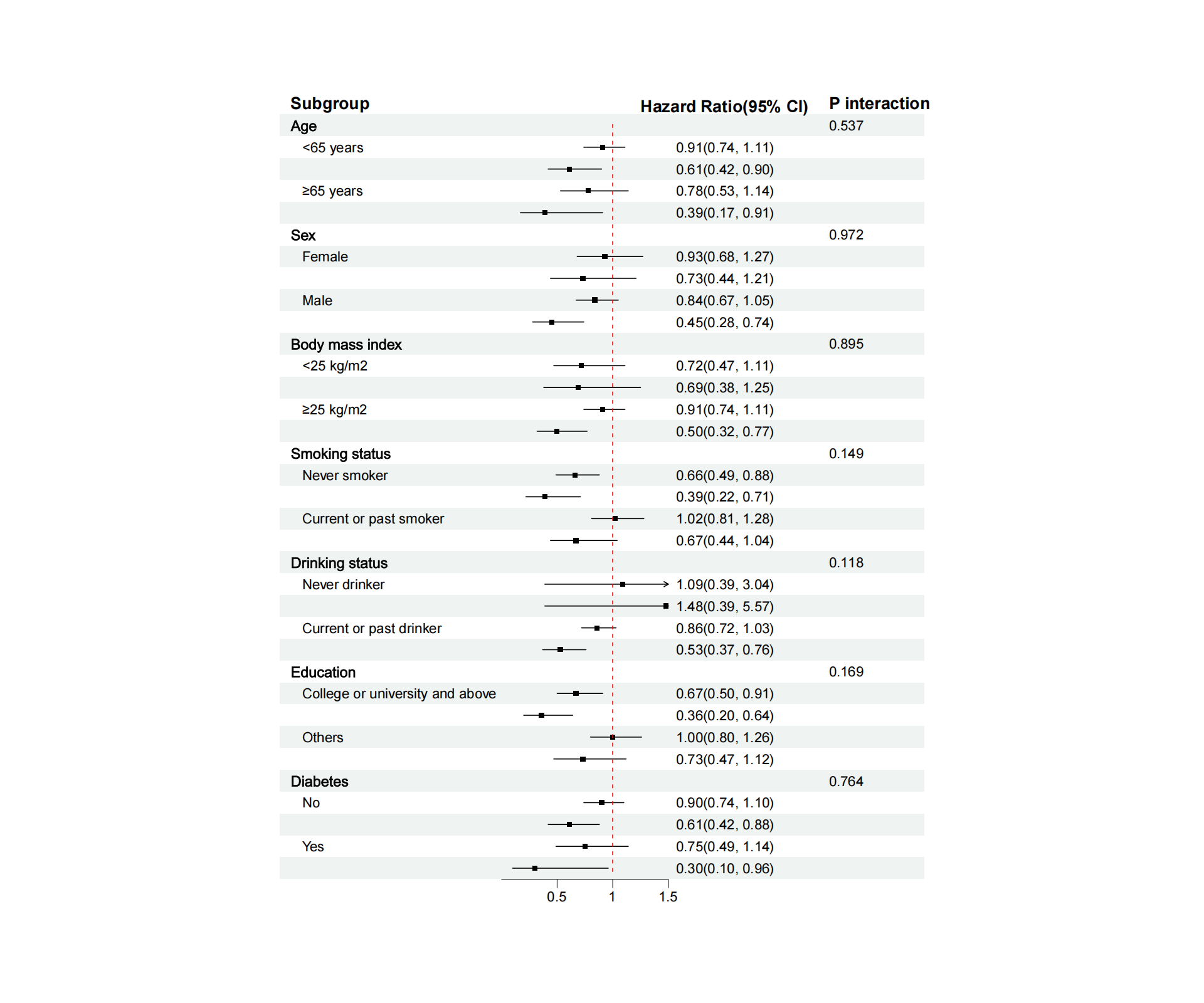


**Figure S5.** Stratified analyses of association between the EAT-Lancet diet index and risk of cirrhosis. *P* for interaction (the likelihood ratio test). Levels of significance: *P* < 0.05. Multivariable Cox proportional hazard models were adjusted for age, sex, ethnicity, Townsend deprivation index, educational level, BMI, physical activity, drinking status, smoking status, household income, CVD, cancer and diabetes.

Abbreviation: HR, hazard ratio; CI, confidence interval; CVD, Cardiovascular Disease.


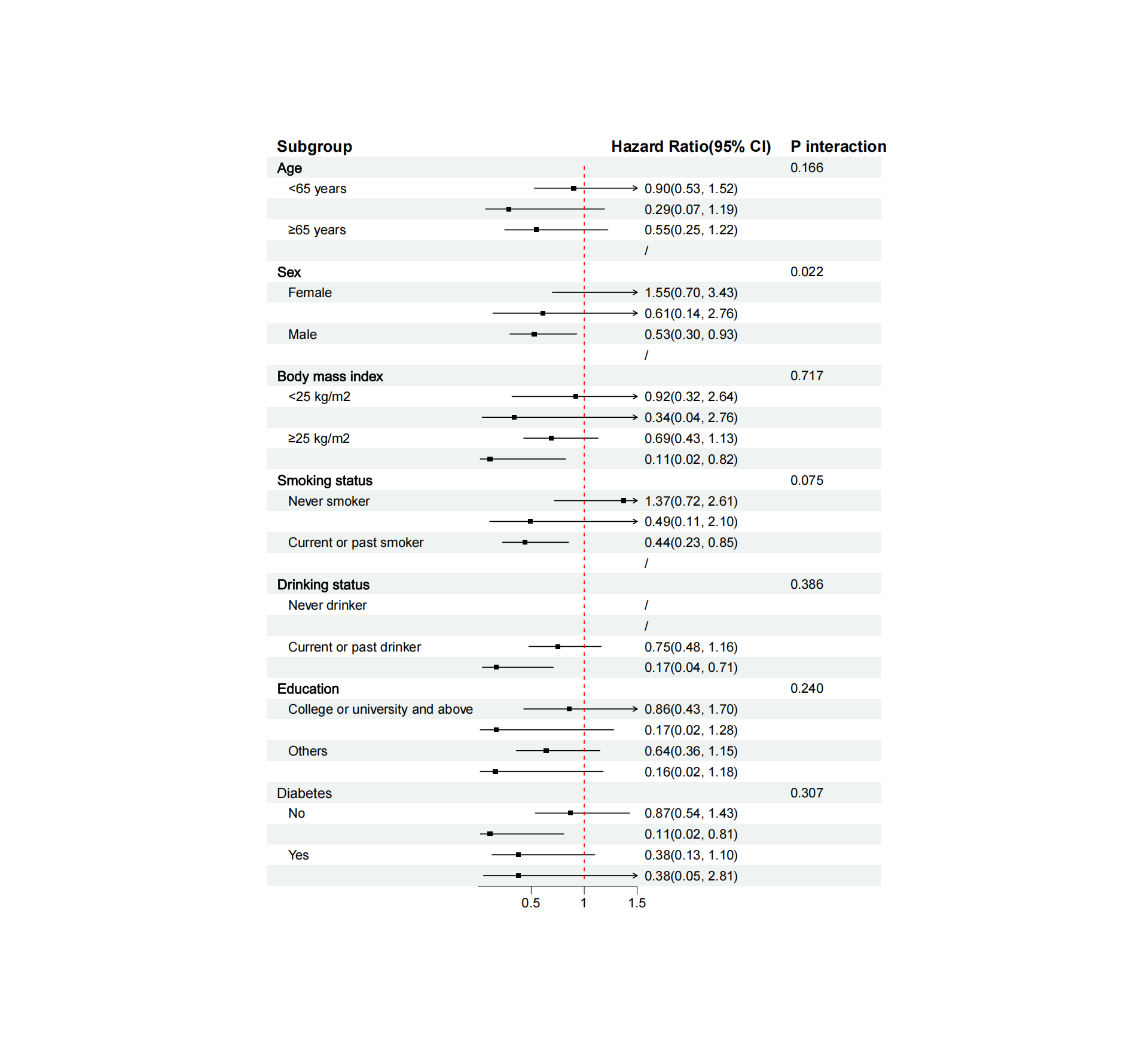


**Figure S6.** Stratified analyses of association between the EAT-Lancet diet index and risk of liver cancer. *P* for interaction (the likelihood ratio test). Levels of significance: *P* < 0.05. Multivariable Cox proportional hazard models were adjusted for age, sex, ethnicity, Townsend deprivation index, educational level, BMI, physical activity, drinking status, smoking status, household income, CVD, cancer and diabetes.

Abbreviation: HR, hazard ratio; CI, confidence interval; CVD, Cardiovascular Disease.


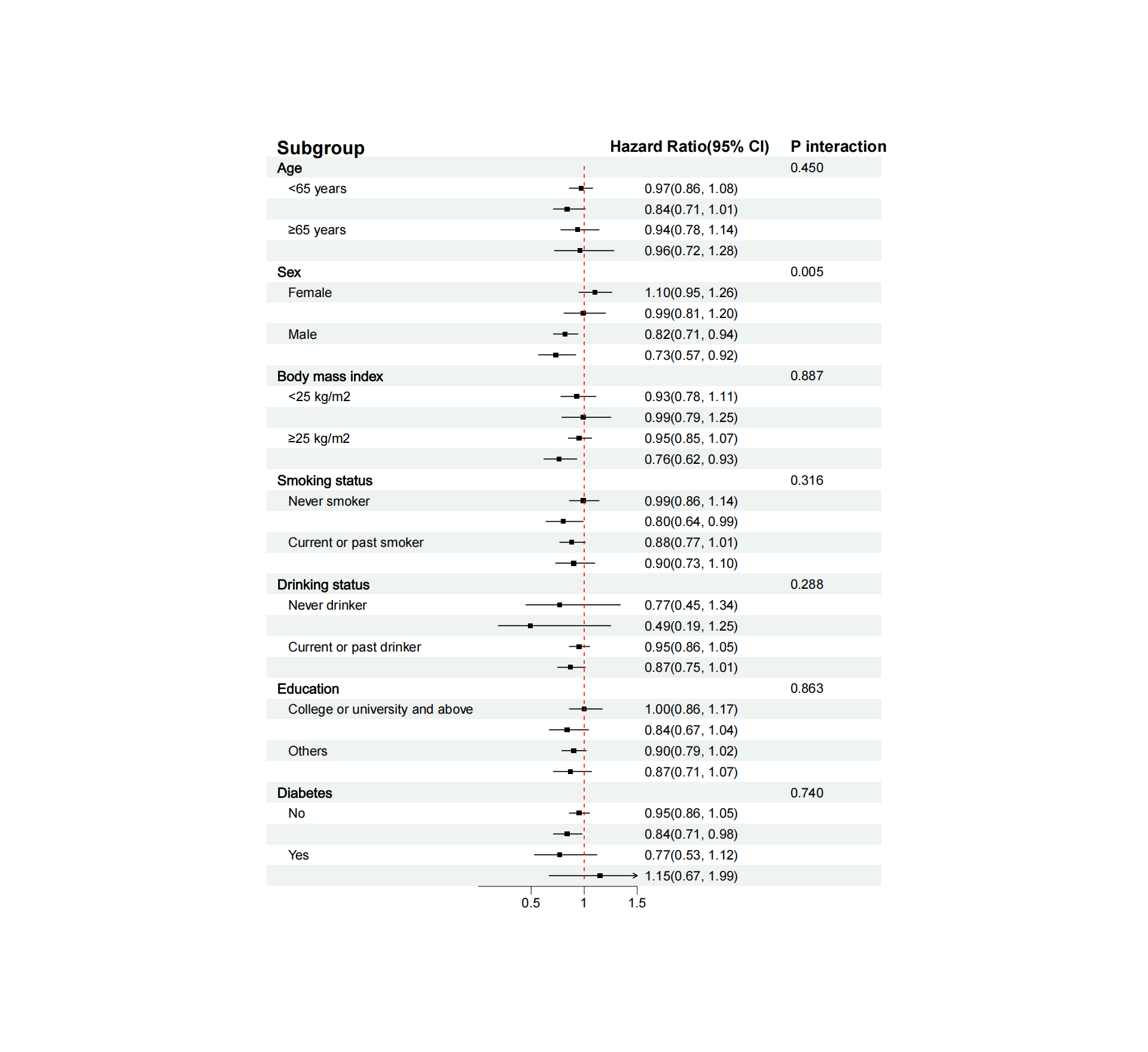


**Figure S7.** Stratified Analyses of Association Between the EAT-Lancet diet index and risk of other liver diseases. *P* for interaction (the likelihood ratio test). Levels of significance: *P* < 0.05. Multivariable Cox proportional hazard models were adjusted for age, sex, ethnicity, Townsend deprivation index, educational level, BMI, physical activity, drinking status, smoking status, household income, CVD, cancer and diabetes.

Abbreviation: HR, hazard ratio; CI, confidence interval; CVD, Cardiovascular Disease.

| **Table S1**. Description of the components of the EAT-Lancet reference diet. | | |
| --- | --- | --- |
| **Dietary Components** | **Criteria for scoring 1 point based on the healthy reference diet** | **Food items based on the Oxford WebQ questionnaire** |
| **Whole grains** |  |  |
| 1. Rice, wheat, corn, and other | ≤464g/day and whole grain fiber >5 grams | porridge, muesli, oat crunch, sweetened cereal, plain cereal, bran cereal, whole-wheat cereal, other cereal, white pasta, wholemeal pasta, white rice, brown rice, sushi, snackpot, couscous, other grain |
| **Tubers and starchy vegetables** |  |  |
| 1. White potatoes and sweet potatoes | ≤100g/day | fried, boiled, baked or mashed potatoes, sweet potato |
| **Vegetables** |  |  |
| 1. All vegetables | ≥ 200g/day | quorn, mixed vegetable, vegetable pieces, coleslaw, side salad, avocado, broad bean, green bean, beetroot, broccoli, butternut squash, cabbage, carrot, cauliflower, celery, courgette, cucumber, garlic, leek, lettuce, mushroom, onion, parsnip, pea, sweet pepper, spinach, fresh tomato, tinned tomato, turnip, watercress, other vegetable, sweetcorn |
| **Fruits** |  |  |
| 1. All fruits | ≥ 100g/day | stewed fruit, prune , dried fruit, mixed fruit, apple, banana, berry, cherry, grapefruit, grape, mango, melon, orange, satsuma, peach, pear, pineapple, plum, other fruit |
| **Dairy foods** |  |  |
| 1. Whole milk or derivative equivalents | ≤ 500g/day | milk, favoured milk, yogurt , low fat hard cheese, hard cheese, soft cheese, blue cheese, low fat cheese spread, cheese spread, cottage cheese, feta, mozzarella, goat’s cheese, other cheese |
| **Protein sources** |  |  |
| 1. Beef, lamb, pork | ≤ 28g/day | bacon, ham, liver, sausage, beef, pork, lamb |
| 1. Chicken, other poultry | ≤ 58g/day | crumbed or deep-fried poultry, poultry |
| 1. Eggs | ≤ 25g/day | whole egg, omelette, eggs in sandwich, scotch egg, other egg |
| 1. Fish | ≤ 100g/day | shellfish, tinned tuna, oily fish, breaded fish, battered fish, white fish, other fish |
| Legumes |  |  |
| 1. Dry beans, string beans, peas | ≤ 100g/day | dried lentils, peas and baked beans |
| 1. Soy foods | ≤ 50g/day | tofu |
| 1. Nuts and seeds | ≥ 25g/day | unsalted peanuts, salted nuts, unsalted nuts, seed |
| **Added fats** |  |  |
| 1. Saturated, mono-unsaturated, poly-unsaturated fatty acid |  | Ratio of 0.8 for unsaturated: saturated fat acid intake |
| **Added sugars** |  |  |
| 1. All sweeteners | ≤ 31g/day | derived from sweet foods and beverages, and sugar added to food or beverages recorded in the 24-hour diet recall |

| Table S2. Genetic variants associated with MASLD. | | | | | | | |
| --- | --- | --- | --- | --- | --- | --- | --- |
| **SNP** | **Chr** | **Pos** | **EA** | **NEA** | **Beta** | **SE** | ***P*-value** |
| rs10201587 | 2 | 202202791 | G | A | -0.044 | 0.006 | 4.18E-12 |
| rs10433937 | 4 | 88230100 | G | T | -0.081 | 0.008 | 5.28E-26 |
| rs1047891 | 2 | 211540507 | A | C | 0.037 | 0.007 | 2.76E-08 |
| rs10774625 | 12 | 111910219 | A | G | 0.039 | 0.007 | 9.75E-09 |
| rs10883451 | 10 | 101924418 | C | T | -0.152 | 0.007 | 2.65E-112 |
| rs11601507 | 11 | 5701074 | A | C | 0.099 | 0.013 | 1.53E-14 |
| rs11621792 | 14 | 24871926 | T | C | 0.042 | 0.007 | 5.48E-10 |
| rs11668950 | 19 | 18282940 | A | G | 0.046 | 0.007 | 2.22E-10 |
| rs11683367 | 2 | 233510011 | C | T | -0.057 | 0.007 | 6.38E-18 |
| rs11683409 | 2 | 112770134 | G | C | 0.044 | 0.007 | 9.19E-12 |
| rs12149380 | 16 | 72043546 | G | C | 0.048 | 0.008 | 2.25E-09 |
| rs12500824 | 4 | 77416627 | A | G | 0.047 | 0.007 | 7.92E-13 |
| rs132665 | 22 | 36564170 | G | A | -0.066 | 0.0098 | 1.18E-11 |
| rs1337101 | 1 | 219726100 | T | G | -0.052 | 0.007 | 1.98E-12 |
| rs148337160 | 10 | 104166504 | C | T | 0.088 | 0.013 | 2.89E-11 |
| rs1547014 | 22 | 29100711 | T | C | -0.064 | 0.0067 | 1.57E-21 |
| rs1626329 | 12 | 121622023 | T | C | 0.05 | 0.007 | 2.61E-14 |
| rs1658943 | 9 | 6676953 | T | C | -0.049 | 0.009 | 3.54E-08 |
| rs17036160 | 3 | 12329783 | T | C | -0.068 | 0.011 | 3.39E-10 |
| rs174535 | 11 | 61551356 | C | T | -0.064 | 0.007 | 1.59E-20 |
| rs17598226 | 4 | 100496891 | G | C | -0.042 | 0.007 | 5.61E-09 |
| rs1801689 | 17 | 64210580 | C | A | 0.17 | 0.019 | 1.46E-18 |
| rs2207132 | 20 | 39142516 | A | G | 0.178 | 0.028 | 1.58E-10 |
| rs2296285 | 13 | 29009673 | A | T | 0.039 | 0.007 | 1.76E-08 |
| rs2642438 | 1 | 220970028 | A | G | -0.075 | 0.007 | 6.65E-24 |
| rs2727324 | 17 | 61922102 | C | G | 0.038 | 0.007 | 1.24E-08 |
| rs2737217 | 8 | 116630311 | A | G | 0.044 | 0.007 | 3.92E-10 |
| rs2943652 | 2 | 227108446 | C | T | -0.062 | 0.007 | 1.04E-19 |
| rs2980888 | 8 | 126507308 | T | C | 0.13 | 0.007 | 4.21E-72 |
| rs340009 | 15 | 60899639 | A | C | 0.043 | 0.007 | 3.65E-11 |
| rs34123446 | 12 | 122511238 | G | A | -0.043 | 0.007 | 2.82E-11 |
| rs35199395 | 10 | 70983936 | G | C | -0.039 | 0.007 | 2.79E-08 |
| rs36086195 | 1 | 16510894 | C | T | -0.043 | 0.007 | 4.50E-10 |
| rs3810367 | 19 | 4342847 | G | T | 0.037 | 0.007 | 3.77E-08 |
| rs3852142 | 5 | 55796968 | T | A | -0.072 | 0.013 | 1.90E-08 |
| rs3935942 | 15 | 73971361 | A | C | 0.055 | 0.007 | 2.72E-15 |
| rs4683438 | 3 | 142652559 | T | G | -0.047 | 0.007 | 4.73E-12 |
| rs4711750 | 6 | 43757082 | A | T | 0.037 | 0.007 | 4.79E-08 |
| rs4734654 | 8 | 103669991 | G | A | -0.044 | 0.007 | 1.55E-10 |
| rs4782568 | 16 | 83980529 | G | C | -0.064 | 0.007 | 8.82E-21 |
| rs4805033 | 19 | 33839554 | G | A | -0.037 | 0.007 | 3.06E-08 |
| rs4841132 | 8 | 9183596 | A | G | 0.123 | 0.011 | 6.62E-32 |
| rs4918722 | 10 | 113947040 | C | T | 0.077 | 0.008 | 1.75E-23 |
| rs5117 | 19 | 45418790 | C | T | -0.072 | 0.008 | 2.21E-20 |
| rs56175344 | 11 | 93864393 | G | C | -0.128 | 0.01 | 4.60E-40 |
| rs6059896 | 20 | 33111783 | C | T | 0.047 | 0.006 | 1.80E-13 |
| rs61791108 | 3 | 170732742 | A | G | 0.111 | 0.019 | 2.73E-09 |
| rs6541349 | 1 | 93787867 | C | T | 0.049 | 0.008 | 1.76E-09 |
| rs6543007 | 2 | 101663584 | T | C | 0.036 | 0.007 | 5.00E-08 |
| rs6734238 | 2 | 113841030 | G | A | -0.057 | 0.006 | 4.94E-19 |
| rs687621 | 9 | 136137065 | G | A | 0.043 | 0.007 | 7.91E-11 |
| rs7041363 | 9 | 117146043 | G | C | -0.13 | 0.007 | 1.02E-81 |
| rs7168849 | 15 | 90346227 | G | A | -0.064 | 0.009 | 1.67E-11 |
| rs73024760 | 2 | 169885122 | T | C | 0.104 | 0.016 | 7.59E-11 |
| rs738408 | 22 | 44324730 | T | C | 0.267 | 0.0076 | 1.00E-200 |
| rs7599 | 19 | 36038390 | A | G | 0.046 | 0.007 | 1.05E-12 |
| rs7653249 | 3 | 136005792 | C | G | -0.076 | 0.008 | 3.51E-22 |
| rs79598313 | 1 | 27284913 | T | C | 0.172 | 0.024 | 4.91E-13 |
| rs799165 | 7 | 73052057 | A | T | 0.06 | 0.01 | 1.32E-09 |
| rs8082024 | 17 | 47945460 | C | T | 0.042 | 0.007 | 2.32E-08 |
| rs8108722 | 19 | 10347084 | T | C | 0.044 | 0.008 | 2.25E-08 |
| rs848559 | 2 | 36694497 | T | A | -0.055 | 0.01 | 1.58E-08 |
| rs934295 | 3 | 149122431 | A | T | 0.058 | 0.007 | 2.93E-16 |
| rs9668670 | 12 | 53278512 | T | A | -0.054 | 0.007 | 2.89E-15 |
| rs146774114 | 12 | 49743142 | A | G | -0.157 | 0.028 | 2.50E-08 |
| rs686250 | 6 | 32585055 | A | G | -0.039 | 0.007 | 3.06E-09 |
| rs74816838 | 1 | 161643560 | T | C | 0.078 | 0.012 | 1.51E-10 |
| rs574044675 | 3 | 172274232 | C | A | -0.235 | 0.026 | 1.96E-19 |
| rs28929474 | 14 | 94844947 | T | C | 0.492 | 0.027 | 9.01E-73 |
| rs13389219 | 2 | 165528876 | T | C | -0.053 | 0.007 | 8.20E-16 |
| rs58542926 | 19 | 19379549 | T | C | 0.209 | 0.013 | 6.54E-62 |
| rs56094641 | 16 | 53806453 | G | A | 0.0415 | 0.0069 | 1.36E-09 |
| rs60315134 | 8 | 8670599 | G | A | 0.0363 | 0.0065 | 2.88E-08 |
| rs138033684 | 6 | 71895252 | G | T | 0.6774 | 0.1119 | 1.42E-09 |
| rs4484649 | 8 | 10571491 | C | A | 0.0449 | 0.0066 | 1.38E-11 |
| rs115038698 | 7 | 87024718 | T | C | 0.6424 | 0.068 | 3.51E-21 |
| rs141505249 | 8 | 145732114 | C | G | -2.0196 | 0.1227 | 7.15E-61 |
| Abbreviations: Chr, chromosome; EA, effect allele; NEA, non-effect allele; Pos: position; SNP; single nucleotide polymorphism; MASLD, metabolic dysfunction-associated steatotic liver. | | | | | | | |

| **Table S3. ICD-10 codes for diseases used as exclusion criteria.** | |
| --- | --- |
| **Disease at or before baseline** | **ICD-10 code** |
| **Other liver diseases** |  |
| Alcoholic liver disease (ALD) | K70 |
| Viral hepatitis | B16, B17, B18, B19 |
| Autoimmune liver disease (autoimmune hepatitis, primary biliary cholangitis, primary sclerosing cholangitis) | K83.0, K74.3, K75.4 |
| Hemochromatosis | E83.1 |
| Wilson | E83.0 |
| Alpha-1-antitrypsin deficiency | E88.0 |
| Budd-Chiari | I82.0, K76.5 |
| Chronic hepatitis, unspecified | K73.9, K73.2 |
| Secondary or unspecified biliary cirrhosis | K74.4, K74.5 |
| **Alcohol/drug use disorder** |  |
| Codes associated with alcohol use disorder | F10 |
| Codes associated with somatic consequences of alcohol (except ALD) | E24.4, G62.1, I42.6, K29.2, G31.2, G72.1, K85.2, K86.0, T51.0, T51.9, Y57.3, X65, Z50.2, Z71.4, Z72.1 |
| Codes associated with drug use disorders except nicotine/caffeine | F11, F12, F13, F14, F16, F18, F19 |
| **Liver transplantation** | Z94.4 |

| **Table S4. ICD-10 codes utilized in the UKB for defining outcomes.** | | |
| --- | --- | --- |
| **Outcomes** | **ICD-10 code** | **Description** |
| MASLD | K76.0 | Fatty (change of) liver, not elsewhere classified |
|  | K75.8 | Other specified inflammatory liver diseases |
| Cirrhosis | K70.2 | Alcoholic fibrosis and sclerosis of liver |
|  | K70.3 | Alcoholic cirrhosis of liver |
|  | K70.4 | Alcoholic hepatic failure |
|  | K74.0 | Hepatic fibrosis |
|  | K74.1 | Hepatic sclerosis |
|  | K74.2 | Hepatic fibrosis with hepatic sclerosis |
|  | K74.6 | Other and unspecified cirrhosis of liver |
|  | K76.6 | Portal hypertension |
|  | I85.0 | Oesophageal varices with bleeding |
|  | I85.9 | Oesophageal varices without bleeding |
| Liver cancer | C22.0 | Liver cell carcinoma |
| Other liver disease | K70.0, K70.1, K70.9 | Alcoholic liver disease |
|  | K71 | Toxic liver disease |
|  | K72 | Hepatic failure, not elsewhere classified |
|  | K73 | Chronic hepatitis, not elsewhere classified |
|  | K74.3, K74.4, K74.5 | Fibrosis and cirrhosis of liver |
|  | K75.0, K75.2, K75.3, K75.4, K75.9 | Other inflammatory liver diseases |
|  | K76.1, K76.2, K76.3, K76.4, K76.5, K76.7, K76.8, K76.9 | Other diseases of liver |
|  | K77 | Liver disorders in diseases classified elsewhere |

| **Table S5.** Association between polygenic risk score and risk of NAFLD | | | |
| --- | --- | --- | --- |
|  | **Low genetic risk** | **Medium genetic risk** | **High genetic risk** |
| Age and sex-adjusted model | 1.00 (Ref.) | 1.11(1.00, 1.22) | **1.20(1.09, 1.33)** |
| Multivariable-adjusted model | 1.00 (Ref.) | **1.17(1.04, 1.32)** | **1.24(1.10, 1.40)** |
| Multivariable-adjusted model further adjusted for ethnicity, Townsend deprivation index, educational level, BMI, physical activity, drinking status, smoking status, household income, CVD, cancer and diabetes. | | | |

| **Table S6.** Association between EAT-Lancet diet index and risk of MASLD, cirrhosis, liver cancer, and other liver diseases, excluding the outcomes of interest within the first two or four years of follow-up | | |
| --- | --- | --- |
|  | **Excluding cases within first 2 y** | **Excluding cases within first 4 y** |
|  | HR(95% CI) | HR(95% CI) |
| **MASLD** |  |  |
| tertile1 | 1.00 (Ref.) | 1.00 (Ref.) |
| tertile2 | **0.82(0.73, 0.91)** | **0.84(0.74, 0.94)** |
| tertile3 | **0.66(0.55, 0.80)** | **0.70(0.57, 0.85)** |
| **Liver cirrhosis** |  |  |
| tertile1 | 1.00 (Ref.) | 1.00 (Ref.) |
| tertile2 | 0.88(0.73, 1.06) | 0.91(0.75, 1.11) |
| tertile3 | **0.56(0.39, 0.80)** | **0.58(0.40, 0.84)** |
| **Liver cancer** |  |  |
| tertile1 | 1.00 (Ref.) | 1.00 (Ref.) |
| tertile2 | 0.76(0.49, 1.17) | 0.80(0.51, 1.25) |
| tertile3 | **0.16(0.04, 0.72)** | **0.19(0.05, 0.76)** |
| **Other liver diseases** |  |  |
| tertile1 | 1.00 (Ref.) | 1.00 (Ref.) |
| tertile2 | 0.96(0.87, 1.06) | 0.97(0.88, 1.08) |
| tertile3 | 0.87(0.75, 1.01) | 0.86(0.73, 1.01) |
| Multivariable Cox models adjusted for age, sex, ethnicity, Townsend deprivation index, educational level, BMI, physical activity, drinking status, smoking status, household income, CVD, cancer and diabetes. | | |

| **Table S7.** Association of EAT-Lancet diet index with MASLD, cirrhosis, liver cancer, and other liver disease using multiple imputation | | | | |
| --- | --- | --- | --- | --- |
|  | **Model1** | | **Model2** | |
|  | HR(95% CI) | *P* value | HR(95% CI) | *P* value |
| **MASLD** |  |  |  |  |
| tertile1 | 1.00 (Ref.) |  | 1.00 (Ref.) |  |
| tertile2 | 0.68(0.62, 0.75) | **<0.001** | 0.79(0.72, 0.87) | **<0.001** |
| tertile3 | 0.53(0.45,0.62) | **<0.001** | 0.70(0.60, 0.82) | **<0.001** |
| Continuous (per 3 unit) | 0.54(0.49, 0.60) | **<0.001** | 0.72(0.60, 0.87) | **<0.001** |
| **Liver cirrhosis** |  |  |  |  |
| tertile1 | 1.00 (Ref.) |  | 1.00 (Ref.) |  |
| tertile2 | 0.74(0.63, 0.86) | **<0.001** | 0.84(0.72, 0.98) | 0.025 |
| tertile3 | 0.51(0.39, 0.86) | **<0.001** | 0.63(0.48, 0.83) | **0.001** |
| Continuous (per 3 unit) | 0.57(0.48, 0.68) | **<0.001** | 0.72(0.60, 0.87) | **<0.001** |
| **Liver cancer** |  |  |  |  |
| tertile1 | 1.00 (Ref.) |  | 1.00 (Ref.) |  |
| tertile2 | 0.69(0.47, 1.01) | 0.057 | 0.77(0.53, 1.14) | 0.189 |
| tertile3 | 0.17(0.05, 0.53) | **<0.01** | 0.21(0.07, 0.66) | **0.008** |
| Continuous (per 3 unit) | 0.41(0.26, 0.64) | **<0.001** | 0.51(0.32, 0.80) | **0.004** |
| **Other liver diseases** |  |  |  |  |
| tertile1 | 1.00 (Ref.) |  | 1.00 (Ref.) |  |
| tertile2 | 0.85(0.79, 0.93) | **<0.001** | 0.91(0.84, 0.99) | 0.029 |
| tertile3 | 0.80(0.71, 0.91) | **<0.001** | 0.88(0.77, 1.00) | **0.044** |
| Continuous (per 3 unit) | 0.86(0.68, 0.84) | **<0.001** | 0.85(0.77, 0.94) | **0.002** |
| Model 1 was adjusted for age and sex.  Model 2 was adjusted for age, sex, ethnicity, Townsend deprivation index, educational level, BMI, physical activity, drinking status, smoking status, household income, CVD, cancer and diabetes. | | | | |
